# High-fertility sows reshape gut microbiota: the rise of serotonin-related bacteria and its impact on sustaining reproductive performance

**DOI:** 10.1186/s40104-025-01191-z

**Published:** 2025-05-22

**Authors:** Yanli Chen, Yan Wang, Weike Shaoyong, Yanmin He, Yalin Liu, Siyu Wei, Yujie Gan, Lu Sun, Youming Wang, Xin Zong, Yun Xiang, Yizhen Wang, Mingliang Jin

**Affiliations:** 1 Institute of Feed Science, College of Animal Sciences, Zhejiang University, Hangzhou, 310058 China; 2 Key Laboratory of Molecular Animal Nutrition, Ministry of Education, Hangzhou, 310058 China; 3 Key Laboratory of Animal Nutrition and Feed Science (Eastern of China), Ministry of Agriculture and Rural Affairs, Hangzhou, 310058 China; 4 Zhejiang Key Laboratory of Nutrition and Breeding for High-Quality Animal Products, Hangzhou, 310058 China; 5 Institute of Animal Husbandry and Veterinary Medicine, Jinhua Academy of Agricultural Science Research, Jinhua, 321017 China

**Keywords:** Gut microbiome, Multi-omics analysis, Reproductive performance, Serotonin

## Abstract

**Background:**

Compelling evidence has established a strong link between the gut microbiota and host reproductive health. However, the specific regulatory roles of individual bacterial species on reproductive performance are not well-understood. In the present study, Jinhua sows with varying reproductive performances under the same diet and management conditions were selected to explore potential mechanisms on the intricate relationship between the gut microbiome and host reproductive performance using 16S rRNA sequencing, metagenomics and serum metabolomics.

**Results:**

Our findings revealed that the KEGG pathways for base excision repair and DNA replication were enriched, along with gene-level enhancements in spore formation, in sows with higher reproductive performance, indicating that the gut microbiome experiences stress. Further analysis showed a positive correlation between these changes and litter size, indicating that the host acts as a stressor, reshaping the microbiome. This adaptation allows the intestinal microbes in sows with high reproductive performance to enrich specific serotonin-related bacteria, such as *Oxalobacter formigenes*, *Ruminococcus* sp. CAG 382, *Clostridium leptum*, and *Clostridium botulinum*. Subsequently, the enriched microbiota may promote host serotonin production, which is positively correlated with reproductive performance in our study, known to regulate follicle survival and oocyte maturation.

**Conclusion:**

Our study provides a theoretical basis for understanding the interactions between gut microbes and the host. It highlights new insights into reassembling gut microbiota in sows with higher litter sizes and the role of serotonin-related microbiota and serotonin in fertility.

**Graphical Abstract:**

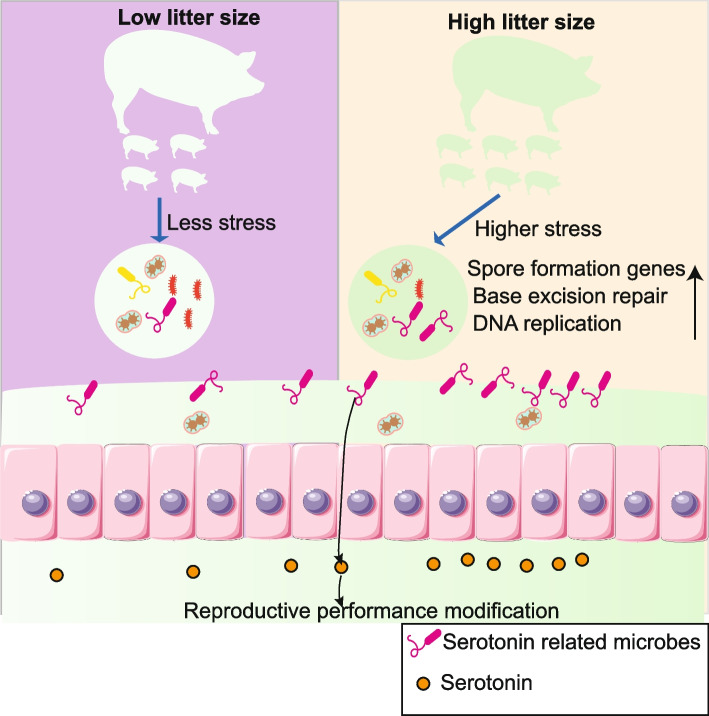

**Supplementary Information:**

The online version contains supplementary material available at 10.1186/s40104-025-01191-z.

## Introduction

The commensal gut microbiota plays a vital role in maintaining host health and significantly contributes to various health effects, including metabolic disease, neurological disorders, infection, and an enhanced immune response [1]. Recognized as a full-fledged endocrine organ, the gut microbiota exerts far-reaching influences on distant organs and numerous metabolic pathways [2, 3]. Recently, there has been a surge in concern over the microbiota’s crucial role in reproductive health. These complex regulatory interactions hinge not only on the delicate direct balance of hormones and metabolic processes essential for the functioning of reproductive organs, but also extend to indirectly modulating wider physiological pathways that are integral to the overall host health level, including immunity, energy balance, and various metabolic pathways [4]. These pathways play a vital role in ensuring optimal host fertility.

The imbalance in these metabolites produced by the gut microbiome and host, along with changes in immune response, energy intake and metabolic balance are identified as significant risk indirect factors impacting reproductive health [5]. The microbiome influences reproductive performance, affecting both males and females. In males, it impacts sperm motility and fertilization, while in females, it plays a role in follicle and oocyte maturation in the ovary, embryo migration, implantation, and the regulation of estrogen, androgens, and other hormones [6]. The perturbations in the gut microbiota can lead to reproductive pathologies [3]. Notably, it was reported that intestinal bacteria play a significant role in estrogen metabolism, as the microbiota can secret β-glucuronidase to metabolize estrogens from conjugate forms to deconjugated form, attributing to active estrogen levels. The most classic example is polycystic ovary syndrome (PCOS) accompanied with the disorder of gut microbiota, higher level of androgens and lower estrogen [7]. Additionally, *Ruminococcus* has been reported to be significantly increased in neonatally androgenized rats and is positively correlated with serum testosterone levels [8]. This evidence further emphasizes the profound influence of gut microbiota on hormonal balance and its potential contribution to reproductive wellness. In addition, it’s important to note that the host’s microbiota [9], especially in the gut, fluctuates during different phases of childbirth, highlighting the complex inter-regulatory relationship between the gut microbiota and reproductive health of host [10].

Despite considerable progress in elucidating the microbiome’s role in reproductive biology, current research on effectively addressing the specific reproductive challenges encountered within the swine industry was limited. Given the increasing demand for improved sow fertility and reproductive efficiency, there is a pressing need to investigate how the gut microbiome influences reproductive physiology under practical production conditions. Notably, sows of the same breed exhibit considerable variation in reproductive capacity, even when provided with identical diets and raised under the same environmental conditions [11]. This variability suggests that factors beyond genetics and management practices contribute to reproductive differences, highlighting the potential role of the gut microbiome [12]. Further investigation into the role of specific microorganisms in regulating host physiology, particularly in relation to hormone regulation and key metabolic pathways essential for reproduction, could yield critical insights for improving sow fertility. A deeper understanding of these mechanisms may contribute to the development of microbiome-based strategies aimed at enhancing reproductive performance and overall productivity in commercial swine production.

In the current investigation, Jinhua sows with varying reproductive performance were selected as subjects. Through comprehensive multi-omics studies, a significant elevation of serotonin level in highly fertile sows were revealed. Specifically, serotonin plays a crucial role in regulating reproductive physiology, including ovarian function, follicular development [11]. Notably, an increased percentage of specific gut microbiota associated with serotonin regulation was also observed in these sows and those changes in the microbiota were related to the total litter size, underscoring a substantial correlation between gut microbiota composition and reproductive performance. This value of exploration not only provides new insights into the influence of gut microbiota on sow reproductive health but also highlights the potential role of the host in shaping its microbiota. These findings lay the foundation for further research on how gut microbiota impacts sow fertility, offering potential strategies to improve reproductive efficiency and productivity in commercial swine production.

## Materials and methods

### Animals and sample collection

This experiment followed guidelines approved by the Animal Welfare Committee of Zhejiang University (Approval No.: ZJU20200161). A total of 120 Jinhua sows, raised in Dayanhe (Zhejiang, China) without bacterial or viral infections, were selected based on their good health status and fed under uniform conditions, including diet, water, and environment. The pigs were fed with Twin feed (Hunan, China). The sows were selected based on specific reproductive performance metrics outlined in Table S1, key criteria included total litter size, litter size per litter, the number of healthy piglets per litter and parity. Healthy piglets were defined by their birth weight, absence of malformations or diseases, active behavior, and survival beyond a defined period. Additionally, the proportion of stillbirths, mummified piglets, and weak piglets were taken into consideration to ensure the selection of sows with optimal reproductive performance. They were divided into three groups: 40 sows with high reproductive performance (High group, HRP), 40 sows with medium reproductive performance (Medium group, MRP), and 40 sows with low reproductive performance (Low group, LRP). We categorized the sows into three groups based on the number of healthy piglets per litter and performed the Principal Component Analysis (PCA). Subsequently, 31 sows from the HRP and LRP groups were randomly selected for to more effectively explore the relationship between gut microbiota and reproductive performance. Fresh feces from all 62 sows were collected into sterile centrifugal tubes and immediately frozen in liquid nitrogen [13, 14] and stored at −80 °C for subsequent analysis. Fresh feces were collected immediately after farrowing. Approximately 10 mL of blood from the ear vein was collected using blood sample collection tubes containing silica and centrifuged at 3,000 × *g* for 15 min at 4 °C. The supernatant was then immediately stored at −20 °C for further serum metabolite analysis.

### 16S rRNA sequencing of fecal bacteria

Total DNA was extracted from the fecal samples (High group: 31, Low group: 31) using the QIAamp DNA Stool Extraction Kit (Qiagen, Germany), following the manufacturer’s protocol. The V3–V4 variable region 16S rRNA gene of bacteria was amplified using barcoded primers (341F: 5′-CCTACGGGNGGCWGCAG-3′; 805R: 5′-GACTACHVGGGTATCTAATCC-3′) [15]. The PCR amplification cycle was performed as followed: initiation at 95 °C for 5 min, 20 cycles of 95 °C for 30 s, 55 °C for 30 s, 72 °C for 30 s, and a final extension of 72 °C for 10 min. The DNA concentration was measured with a Nanodrop-2000 (Thermo Fisher Scientific, USA), and purified PCR products using AMPure XT Beads (Beckman Coulter Genomics, Danvers, MA, USA) were used to prepare sequencing libraries using the TruSeq Nano DNA LT Library Preparation Kit (FC-121-4001). The amplicons were then purified and pooled for paired-end sequence on an Illumina MiSeq platform (Illumina, San Diego, CA, USA) by Majorbio Company (China) following the standard protocols.

High-quality reads with a rRNA sequence similarity ≥ 97% were defined as an operational taxonomic unit (OTU) using UCLUST (v 1.2.22) [16], while sequences shorter than 110 nucleotides or sequences with an overlap of less than 10 bp were removed. Chimeric sequences were screened and discarded using Usearch (v 8.1.1). The taxonomy of each 16S rRNA gene sequence was analyzed by UCLUST against the Silva 16S rRNA database (v138) using a confident threshold of 90% [17]. Finally, an OTU table was produced for abundance information of microbial taxa and further analysis.

Bacterial community profiles of sows were compared using MicrobiomeAnalyst 2.0 (https://www.microbiomeanalyst.ca/). Chao, Shannon, Simpson and ACE indices were used to analyze the alpha diversity (α-diversity) of fecal bacterial communities generated from High and Low reproductive performance sows, and differences were compared using one-way ANOVA. Beta diversity (β-diversity) analysis was conducted by principal coordinate analysis (PCoA) using Bray–Curtis distance matrices [18]. Relative abundance at different taxa levels was visualized using R ggplot2 package (R 4.3.3). Differentially abundant taxa were identified using the Wilcoxon test, statistical significance was set at adjusted *P* < 0.05.

### Metagenomics sequencing of fecal microbiome

DNA was then fragmented to an average size of approximately 400 bp using the Covaris M220 system (Majorbio Company, China) for paired-end library construction. Individual sequencing libraries were prepared using the TruSeq Nano DNA Library Preparation Kit. Metagenome libraries were sequenced on an Illumina NovaSeq 6000 platform (150 bp paired-end sequencing; Illumina Inc., Hangzhou, China). The 3′ and 5′ adapter sequences were removed from the paired-end reads and single-end reads using fastp. Low-quality reads (quality scores < 18 or length < 50 or containing N bases) were filtered out using fastp. Host reads were filtered by aligning reads with the Burrows-Wheeler-Alignment Tool (BWA, v 0.7.9a) [19] and removing contaminant reads with high-scoring alignments. High-quality reads were assembled into contigs using Multiple Megahit [20]. Open reading frames (ORFs) were predicted from the obtained contigs using Prodigal [21]. Non-redundant genes were identified using CD-HIT at ≥ 90% sequence identity and ≥ 90% coverage [22]. Quality-filtered sequence reads (500/300 bp) were mapped to the representative sequences with > 95% identity, and the total gene abundance in each sample was calculated using SOAPaligner (v 2.2.1) [23].

Representative sequences of the non-redundant gene catalog were aligned to the National Center for Biotechnology Information (NCBI) NR database using BLAST (v 2.2.28) and taxonomic annotations were performed using DIAMOND (v 0.9.14) based on the RefSeq database. Microbial abundance profiles with a relative abundance ≥ 0.01% in more than 50% of all samples were used for gut microbiota analysis. Annotation of gut microbial functions was conducted using Diamond against Kyoto Encyclopedia of Genes and Genomes (KEGG) database (v 94.2). The abundance of KEGG pathways was normalized to transcripts per million (TPM), and pathways with > 5 TPM in at least 50% of all samples were used for biological function analysis [24]. Carbohydrate-active enzyme (CAZyme) annotation was performed using hmmscan against the CAZyme database (v 5.0) [25]. All these databases had an E-value cut-off (1e^−5^) while annotating ORFs.

The α-diversity of fecal bacterial and microbiota communities generated from High and Low reproductive performance sows was analyzed using Chao, Shannon, Simpson and ACE indices. Differences were compared using one-way ANOVA. Visualization of bacterial community structure differences within taxonomical level based on β-diversity was conducted using the PCoA method via the R package. Linear discriminant analysis effect size (LEfSe) analysis was employed to identify significantly differential bacterial species between the two groups. The logarithmic Linear discriminant analysis (LDA) score threshold was set to 2 for biomarker identification and further correlation analysis. To further visualize interaction network diagram difference among microorganisms in the HRP and LRP group individually, the correlation analysis of the top 100 taxa among metagenomic species based on their relative abundance was performed by Spearman’s correlation analysis using Rstudio. Then we visualized the correlation network graph using Gephi (v.0.9.1) software (*P* < 0.05, Spearman’s |*r*|> 0.7). The correlation between the different microbiotas based on LEfSe analysis and other reproductive performances (Table S1) was performed using the website ChiPlot.

### Metabolite extraction, profiling and analysis

The plasma samples from High (*n* = 10) and Low (*n* = 8) groups were immediately placed on ice. Each sample (20 μL) was extracted with 120 μL 50% methanol, vortexed for 1 min, and then incubated at room temperature for 10 min. The extraction mixture was then stored at −20 °C for 12 h. After centrifugation at 4,000 × *g* for 20 min, the supernatants were transferred into new 96 well plates [26], and the extracted metabolites were stored at −80 °C prior to the liquid chromatography mass spectrometry (LC-MS) determination. The intensity of peak data was processed by metaX [26]. Features were assessed in less than 50% of quality control (QC) samples, and the remaining peaks with missing values were imputed with the k-nearest neighbor algorithm to further improve the metabolites quality [27]. The raw dataset was then analyzed using MetaboAnalyst 5.0. Peak areas were normalized using internal standards. Metabolites exhibiting a percentage relative standard deviation (SD) > 30% in the QC samples were excluded, and the normalized data was analyzed. To examine metabolic profile differences among the two groups, PCoA and orthogonal partial least squares discriminant analysis (OPLS-DA) were employed [28]. Significant differential metabolites among the two groups were identified based on a variable importance in projection (VIP) value exceeding 1, a fold change (FC) value exceeding 2, and *P* < 0.05 (Wilcoxon rank-sum test) [29]. The heatmap of the key metabolites of interest among the groups was generated using R heatmap package. The receiver operating characteristic (ROC) curves were generated using the pROC package.

### Bioinformatics and statistics visualization methods

Random forest analysis using the randomForest package in R was employed [30], with serum metabolites as inputs to classify the High or Low group. The contributions of key bacteria to serotonin production were also identified using random forest analysis. Receiver Operating Characteristic (ROC) curve results were plotted using the R pROC package. The ROC curve was constructed, and the area under the curve (AUC) was used to designate the ROC effect [31]. Correlation analysis among metagenomic and metabolomic data was performed using metagenomic species and metabolites identified as significantly different between HRP and LRP samples, incorporating adjustment for sow phenotypic scores. Spearman’s rho was calculated using the corr.test function within the R psych package. Furthermore, Benjamini-Hochberg adjustment for multiple comparisons [32]. A phylogenetic tree was constructed and visualized using meinverse (https://meinverse.cn/). All final Figures were assembled using Adobe Illustrator. The significant bacterial biomarkers identified by random forests regression of relative abundances of LEfSe-determined bacterial taxon (LDA > 2) against serum serotonin concentration using R randomForest package.

## Results

### Jinhua sows have different reproductive performance

**Fig. 1:**
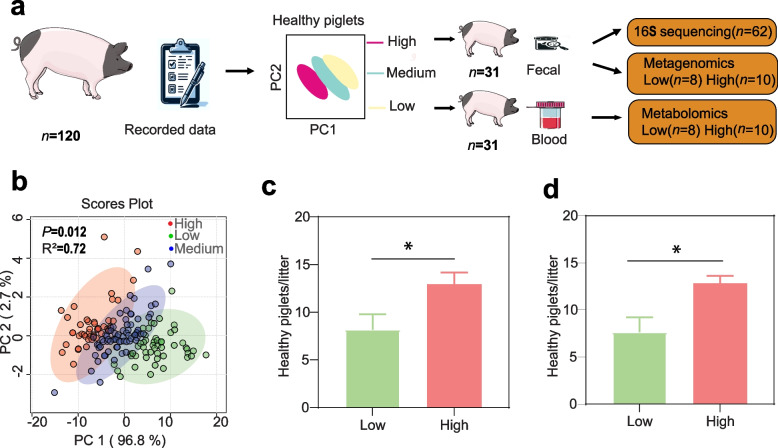
The design of the study and reproductive performance of sows in the study. **a** Experimental design of the study. **b** The score plot according to reproductive performance of sows, (Low reproductive performance, LRP; Medium reproductive performance, MRP, High reproductive performance, HRP). **c** Comparison of healthy piglets per litter between the HRP and LRP groups (*n* = 31). **d** Comparison of healthy piglets per litter between the LRP (*n* = 8) and HRP groups (*n* = 10). A comparison of the number of healthy piglets per litter between the LRP (*n* = 8) and LRP (*n* = 8) was performed using Student’s t-test. Date are presented as the mean ± SD. ^*^P < 0.05 (Student’s *t*-test)

We conducted the present study on 120 Jinhua sows to explore their reproductive performance. To better understand the relationship between gut microbes and reproductive performance, we categorized the sows into three groups based on the number of healthy piglets per litter and performed the PCA as illustrated in Fig. 1a and b. After the initial analysis, we selected 62 healthy Jinhua sows and divided them into two groups, namely HRP and LRP, based on their healthy piglets per litter (Table S1) for fecal 16S rRNA gene amplicon sequencing. Subsequently, we chose 18 samples (LRP: *n* = 8; HRP: *n* = 10) from the initial 62 for fecal metagenome sequencing and metabolomics analysis. To ensure the relevance of our subsequent analyses, we statistically assessed the number difference of piglets per litter between the two groups. The results indicated a notable distinction in piglets per litter between the LRP and HRP groups, with averages of 8.16 ± 1.60 and 12.93 ± 1.23, respectively (Fig. 1c). Additionally, we observed a significant difference with average health piglets per litter with 7.62 ± 1.60 and 12.82 ± 0.80 of LRP and HRP sows for metabolomics and metagenomics analysis (Fig. 1d). Further reproductive profiles of each sow are detailed in Table S1.

### Fecal microbiota difference between HRP and LRP sows using 16S rRNA gene sequencing

**Fig. 2:**
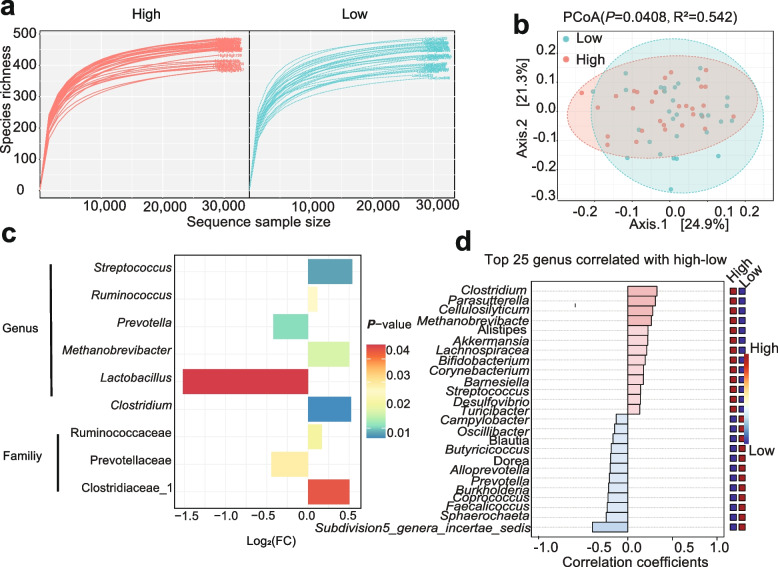
The fecal microbiota of HRP and LRP sows (*n* = 31) was distinguished using 16S rRNA gene amplicon sequencing. **a** Rarefaction Curve analysis based on sequence sample size. **b** Bacterial compositional profiles of HRP and LRP groups visualized using PCoA based on Bray-Curtis distance at the genus level. **c** Significantly different bacterial taxa tested by Wilcoxon test (HRP vs. LRP). **d** Correlation analysis identifying the fecal microbes most associated with high and low reproductive performance between the HRP and LRP groups using Spearman’s rank correlation

To compare the gut bacterial community composition between HPR and LPR groups, 16S rRNA gene sequencing was initially conducted. It was found that there was no significant difference in fecal microbiota α-diversity (*P*_ACE_ = 0.303, *P*_Chao_ = 0.302, *P*_Shannon_ = 0.221 and *P*_Simpson_ = 0.542), as assessed by the Wilcoxon rank-sum test, which suggests that species richness and evenness were similar in the different cohorts (Fig. S1a). The β-diversity between microbiota samples, as determined by PCoA analysis, showed that two clusters were relatively separated, indicating the distinguished bacterial structures between groups (Fig. 2b, *P* = 0.0408). The Rarefaction Curve of bacterial communities between the two groups of sows were shown in Fig. 2a. For the downstream comparison of fecal bacterial taxa, it was found that Firmicutes/Bacteroidetes were the most abundant phyla, with a combined average abundance of over 85% (Fig. S1b). In addition, the percentage of gut beneficial species (*Clostridiaceae_1*, *Ruminococcaceae* and *Streptococcaceae*) of Jinhua sows with higher reproductive performance were higher at the family level, with a reduction of relative abundance of *Prevotellaceae* compared with LRP group. The enriched and decreased families showed a highly variable trend between the two groups (Fig. S1c). At the genus level, significantly variations of different genera were observed between the two groups. As indicated in Fig. 2c and Fig. S1d, *Clostridum*, *Streptococcus*, *Methanobrevibacter*, and *Ruminococcus* were significantly increased in HRP sows, while *Prevotellacea* markedly reduced. Additionally, *Clostridum*, *Parasutterella*, *Methanobrevibacte*, and *Cellulosilyticum* were identified as core gut microbes in the HRP group related to high reproductive performance, while *Subdivision5_genera_incertae_sedis* was recognized as the core gut microbe in the LRP group (Fig. 2d). In conclusion, a significant difference in overall bacterial community composition was observed between HPR and LPR sows’ gut microbiota.

### Differential fecal microbiota and taxonomic indicators through metagenomic sequencing

**Fig. 3:**
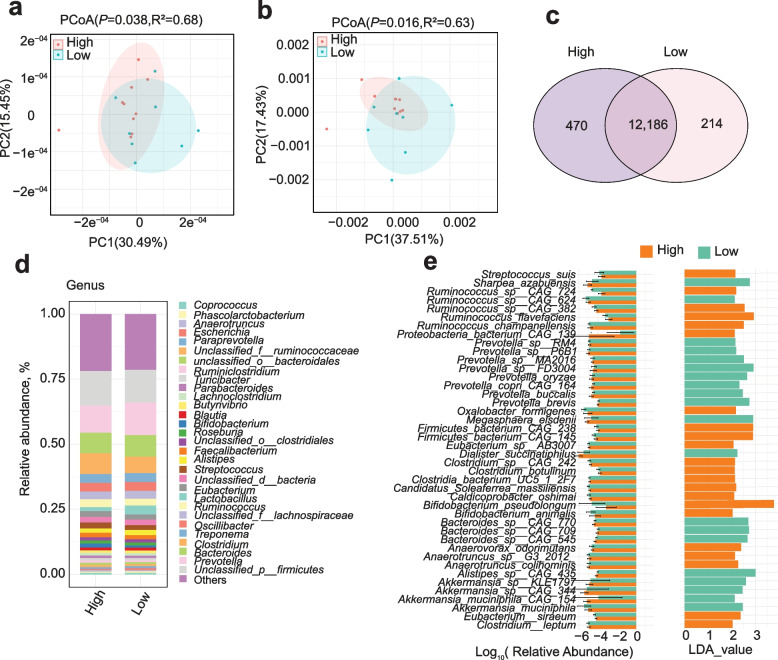
Distinct fecal microbiota of HRP (*n* = 10) and LRP sows (*n* = 8) analyzed using metagenome sequencing. **a** and **b** Microbiota compositional profiles of HRP and LRP sows visualized using PCoA individually based on Bray-Curtis distance on genus and species levels. **c** Venn diagram analysis based on different groups of gut microbes at the species level. **d** The relative abundance of microbial composition at the genus level. **e** LDA score plot of enriched bacterial taxa abundance determined by LEfSe (LDA value > 2.0;* P* < 0.05)

Following the identification of distinct HRP-associated fecal taxa using 16S rRNA gene sequencing, we aimed to enhance the resolution of these findings through metagenomic sequencing, allowing us to unveil more detailed insights into interactions of the microbial communities involved. Consistent with the 16S rRNA gene sequencing analysis, HRP and LRP samples could be distinguished at the genus and species level (Fig. 3a and b, *P* = 0.038, *P* = 0.016, respectively, Bray-Curtis distances), despite considerable variation in community composition between individuals and no significant differences in α-diversity between the groups (Fig. S2a). In addition, Veen diagram analysis further demonstrated difference at the genus (Fig. S2b) and species (Fig. 3c) levels. At the genus level, the HRP group exhibited increased levels of *Clostridium*, *Ruminoccus*, *Ruminostridum* and *Streptococcus* (Fig. 3d), consistent with the findings from the 16S rRNA gene sequencing analysis. Notably, it has been reported *Clostridia* are the main triggers of sporulation [33, 34], which may be related to serotonin synthesis. To identify species contributing to the distinction between HRP and LRP samples, we employed LEfSe analysis to identify significantly different species and the largest difference between the two groups respectively (Fig. 3e, Fig. S2c). The results indicated that several species, including *Bifidobacterium pseudolongum*, *Caldicoprobacter oshimai*, *Clostridium leptum*, *Clostridium botulinum*, *Eubacterium siraeum*, *Ruminococcus champanellensis*, *Ruminococcus flavefaciens*, *Ruminococcus* sp. CAG 382, *Oxalobacter formigenes*, *Anaerovorax odorimutans*, *Methanobrevibacter olleyae* were enriched in the HRP group. Consistent with the above results, the enriched bacteria mainly belong to the genera *Clostridium* and *Ruminococcus*, which are typically associated with host health maintenance and immunity [35]. We then analyzed short-chain fatty acid (SCFA) biosynthesis pathway and genes encoding enzymes associated with SCFA biosynthesis (Fig. S2d). However, there were no differences in CAZyme genes involved in SCFA biosynthesis enriched in the HRP group (Fig. S2d).

**Fig. 4:**
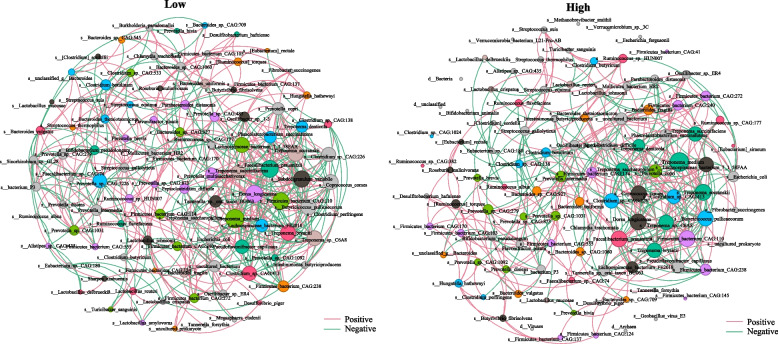
The correlation network of top 100 gut microbiota in the LRP and HRP groups based on metagenome (*P* < 0.05, Spearman’s |*r*|> 0.7, Tables S5 and S6)

Subsequently, we delved into the study of intestinal bacterial communities by conducting an extensive correlation network analysis of the 100 most abundant microbes in each group, revealing these taxa differences of correlation between two groups. As depicted in Fig. 4, it is evident that the correlation between core bacteria, including *Treponema socranskii*, *Faecalibacterium prausnitzii*, *Treponema bryantii*, and *Treponema medium*, had changed. The networks of some key species identified through LEfSe analysis also display significant alterations. For instance, *Clostridium botulinum*, which was positively correlated with *Prevotella* CAG 5226 in the LRP group, was now positively correlated with *Bacterium P3* and *Chlamydia trachomatis* in the HRP group. These findings further elucidate the distinctions in the complex network relationships of gut microbiota between the two groups.

### Serotonin was identified as a key regulator associated with reproductive performance

**Fig. 5:**
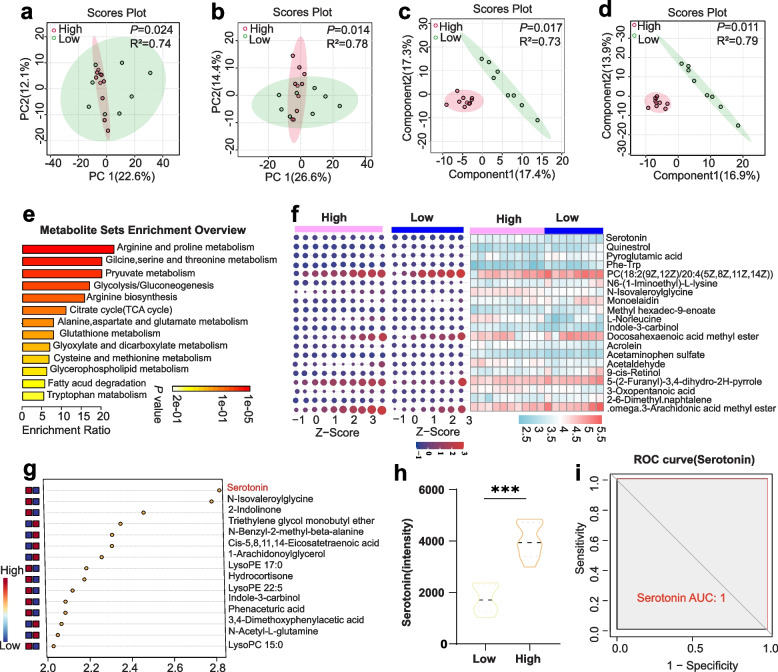
Serum metabolome analysis of the HRP and LPR groups. **a **and **b** PCA of samples based on peaks detected in positive and negative ion modes based on Bray-Curtis distance. **c **and **d** OPLS-DA of samples based on peaks detected positive and negative ion modes based on Bray–Curtis distance. **e** KEGG enrichment analysis of differential metabolites (FC > 1.5, *P* < 0.05, VIP > 1, Table S4). **f** The heatmap based on significantly difference in each sample (FC > 2.0, *P* < 0.05, VIP > 1), with data centered with log_10_ transformed intensity of metabolites. **g** Random forest model for biomarker identification. **h** The difference of serotonin between the HRP and LRP groups. ^***^P < 0.001. **i** Receiver Operating Characteristic (ROC) curve analysis of serotonin for individual metabolite biomarkers

Natural serum components, including metabolites, play a pivotal role in the reproductive performance of the pigs [36, 37]. To further explore the metabolic difference between the HRP and LRP groups, we conducted untargeted metabolomic profiling of unpaired serum samples, identifying 4,740 and 4,011 annotated high-quality feature compounds in positive and negative ion modes, individually. Additionally, there were 523 and 312 of Mass Spectrometry2 (MS2) key metabolites in positive and negative ion modes, individually, which were used in the downstream analysis, as detailed in Table S2. Global examination of MS2 metabolites using PCA showed distinct serum metabolic profiles of HRP and LRP samples in positive and negative ion modes (Bray-Curtis distance) (Fig. 5a and b). Our study revealed a significant and complete separation of the two different reproductive performance samples both in positive and negative ion mode. Notably, the negative and positive metabolites were clearly distinguished between the LRP control and HRP groups based on OPLS-DA (Fig. 5c and d). This distribution demonstrated significant degree of separation between the two groups. Furthermore, 51 significant metabolites were screened for KEGG pathway enrichment analyses by the OPLS models and fold change analysis (HPR vs. LPR, FC > 1.5, *P* < 0.05, VIP > 1) (Fig. 5e). These metabolites were classified into three categories by the secondary classification, namely organic acids and derivatives, lipids and lipid-like molecules and benzenoids. Among them, various metabolites were found to be upregulated in the HRP group compared with the LRP group, including D-Malic acid, Ornithine, L-Norleucine, Creatine, Betaine, 3-Oxopentanoic acid, L-Proline, Acrolein, Pyruvic acid, LysoPC 15:0, Hydrocortisone, Serotonin, Gly-Val, and Indole-3-carbinol (Fig. 5f). The significantly different KEGG pathways revealed that the differential metabolites primarily focused on pyruvate metabolism, glycolysis, tryptophan metabolism and citrate cycle metabolism (Fig. 5e). These results indicated that the altered metabolites primarily focused on amino acid metabolite pathways, reflecting a higher energy supply in the individual HRP sow. Additionally, more amino acid metabolic pathways were also enriched, including glycine, serine, threonine, alanine, aspartate and glutamate metabolites.

To further delineate the metabolites contributing to reproductive performance, we plotted heatmaps (Fig. 5f) to reveal the difference in key metabolites based on strict screening criteria between the two groups (FC > 2, *P* < 0.05, VIP > 1), including serotonin, quinestrol, pyroglutamic acid, Phe-Trp, N-Isovaleroylglycine, Monoelaidin, Methyl hexadec-9-enoate, L-Norleucine, Indole-3-carbinol, Docosahexaenoic acid methyl ester, and Acrolein. A random forest model and ROC curve were used to evaluate the discrimination ability of the key metabolite (Fig. 5g). Remarkably, serotonin was found to be significantly higher in HRP sows (Fig. 5h), which is known to be involved in regulating reproductive capacity, and potentially serving as biomarker for increased litter size of sows [38]. The area under the curve (AUC), sensitivity (positive rate), and specificity (true negative rate) were calculated as follows: serotonin (AUC = 1, specificity = 1, positive rate = 0) (Fig. 5i).

### Integration of multi-omics data and reproductive phenotypes

**Fig. 6:**
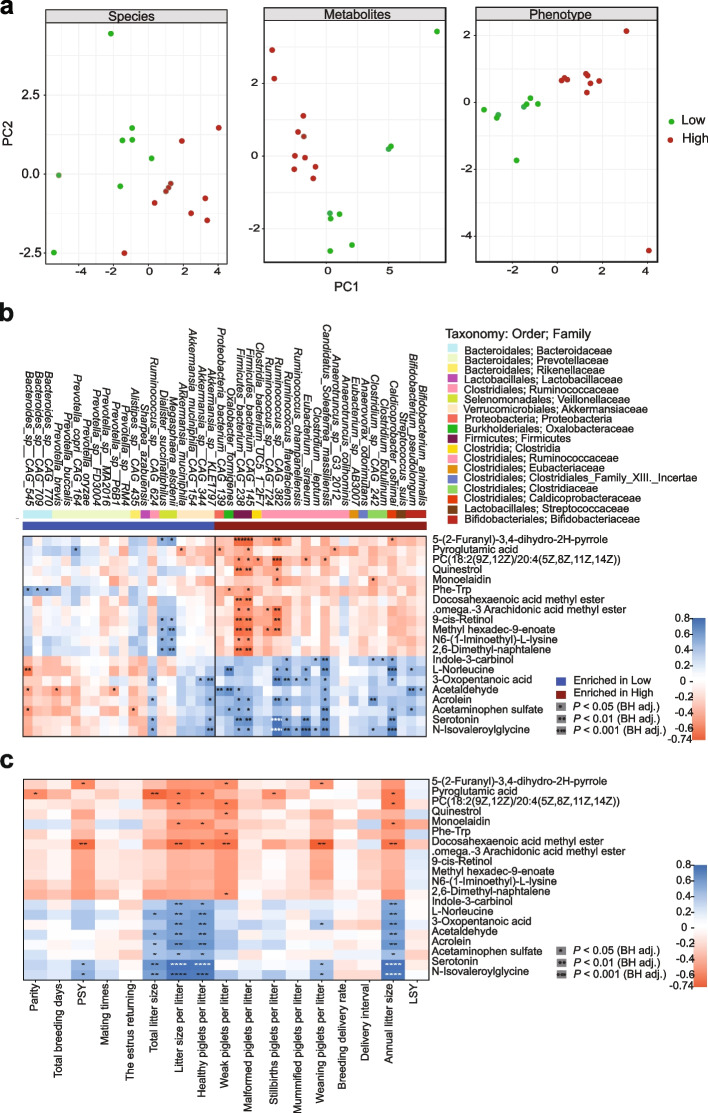
Multi-omics correlation analysis of the fecal microbiota with serum metabolome, reproductive characteristics and serum metabolome. **a** PCA analysis at the species level based on LEfSe analysis (LDA > 2) level, significant metabolites (FC > 2, VIP > 1, *P* < 0.05) and reproductive phenotypes of sows. **b** The association of significantly differential species and metabolites. **c** The correlations of reproductive phenotypes and significantly differential metabolites. Significant correlations denoted by stars (*P < 0.05; **P < 0.01, Benjamini-Hochberg adjustment for multiple comparisons, Table S7)

The mixOmics platform was utilized to explore pivotal species, significant metabolites, and reproductive phenotypes contributing to the differentiation between HRP and LRP samples, integrating these findings into a multi-omic signature (Fig. 6a). It was confirmed that a higher abundance of specific species in HRP sows (Fig. 3e) associated with high fertility.

Subsequently, we conducted an in-depth correlation analysis between gut microbiota, reproductive phenotypes, and serum metabolites to further explore associations between microbial taxa and serotonin, including other metabolites, and the possible influence of these associations in reproductive performance. The phenotypes, different species, metabolites between two groups were separated using PCA analysis (Fig. 6a). The multi-omics correlation analysis revealed that HRP-associated bacteria (including *Ruminococcus* sp. CAG 382, *Firmicutes bacterium* CAG 238, *Firmicutes bacterium* CAG 145, *Oxalobacter formigenes*, *Candidatus Soleaferrea massiliensis*, *Ruminococcus champanellensis*, *Clostridium leptum*, *Clostridium botulinum*) were positively correlated with metrics such as healthy piglets per litter, weaning piglets per litter, and litter size (|*r*|> 0.2, *P* < 0.05; Fig. 6b). Same as above, LRP-associated bacteria (such as *Prevotella buccalis*, *Prevotella brevis*, *Prevotella* sp. RM4) were negatively correlated with these reproductive metrics. A metabolite of tryptophan metabolism, serotonin, exhibited positive correlations with various reproductive phenotypes, including healthy piglets per litter, weaning piglets per litter and litter size (Fig. 6c). Notably, these findings align with existing research indicating that the serotonin system influences reproductive functions in other species, such as fish via the serotonin [38]. These results collectively suggest that the gut microbiota may be associated with reproductive performance by regulating the host’s serotonin levels, thereby furthering reproductive performance in sows.

### Functional and spore formation genes profiling of the gut microbiota between two groups

**Fig. 7:**
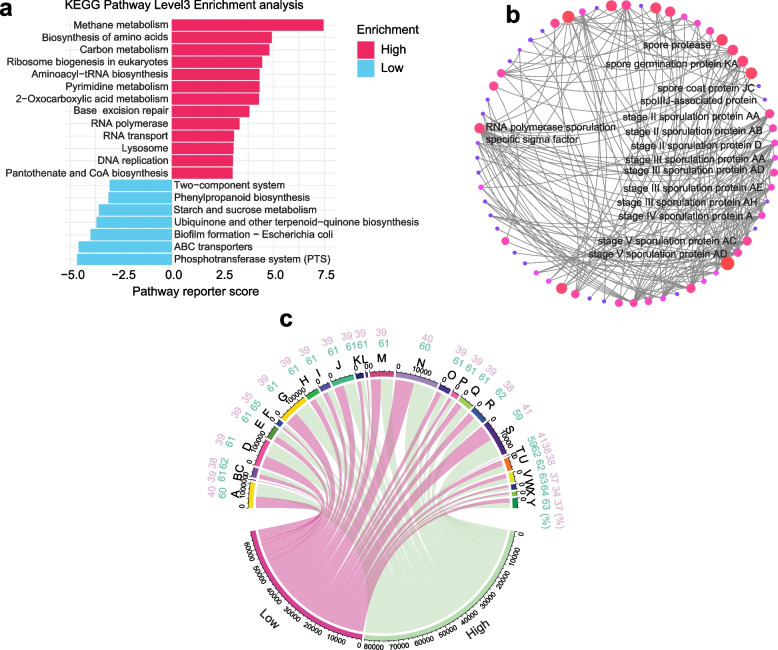
The function and gene difference between two groups. **a** Differentially represented metabolic pathways at the Kyoto Encyclopedia of Genes and Genomes (KEGG) level 3 (*P* < 0.05). **b** Highly correlation network of significantly changed genes of metagenome based on LEfSe analysis (Top 180 of significantly different genes) (*P<*0.05, Spearman’s *r* > 0.9, LDA > 1). **c** Circos plot depicting the abundance significantly changed spore-related genes based on metagenome (Top 25 of significant genes) (*P* < 0.05, Spearman’s *r* > 0.9, LDA > 1) between HRP and LRP groups. Capital letters represent different genes (Table S3)

In light of the differences in the gut microbiota in sows with varying reproductive performance, we conducted a comprehensive functional analysis, as depicted in Fig. 7a. It revealed functional differences in the gut microbiota, including the enrichment of various pathways in the HRP group, such as methane metabolism, pyrimidine metabolism, DNA replication, base excision repair, ribosome biogenesis in eukaryotes, RNA polymerase, aminoacyl-tRNA biosynthesis and lysosome pathways, which indicates that gut microbes was under stressed [35]. Furthermore, biosynthesis of amino acids and methane, 2-Oxocarboxylic acid, histidine, pantothenate and CoA biosynthesis pathways were also enriched. Conversely, in the LRP group, pathways such as phosphotransferase system, starch and sucrose metabolism, ABC transporters, ubiquinone and other terpenoid-quinone biosynthesis pathways are typically associated with energy turnover [39]. Biofilm formation, a major cause of chronic infections, was also observed in LRP group. As carbohydrate metabolism by gut microbes can produce SCFAs, which have been reported to be related to reproductive performance [40], we then analyzed SCFA biosynthesis pathway and genes encoding enzymes associated with SCFA biosynthesis (Fig. S2d). However, our study revealed that there was no difference on ubiquitous genes involved in SCFA synthesis enriched in the HRP group. Furthermore, there were no differences in GH13 involved CAZymes were specifically related with starch and sucrose metabolism (Fig. S2d).

Moreover, a network analysis based on robust and significant correlations (Spearman’s *r* > 0.9) indicated different gene networks predominantly centered on spore-related genes (Fig. 7b), including various stage V sporulation proteins AD and AC, spore germination protein KA, spore coat protein JC, spore protease, stage II sporulation protein AB, stage 0 sporulation protein A, and RNA polymerase sporulation-specific sigma factor. We further visualized the spore formation genes between two groups, observing that the LRP group exhibited a lower relative abundance of spore-related genes compared to the HRP group (Fig. 7c). These findings strongly further suggest that gut microbiota was under stress and had undergone significant changes in sows with high reproductive performance.

### Enhanced litter size in sows drives alterations in gut microbiota and an increase in serotonin-associated bacteria

**Fig. 8:**
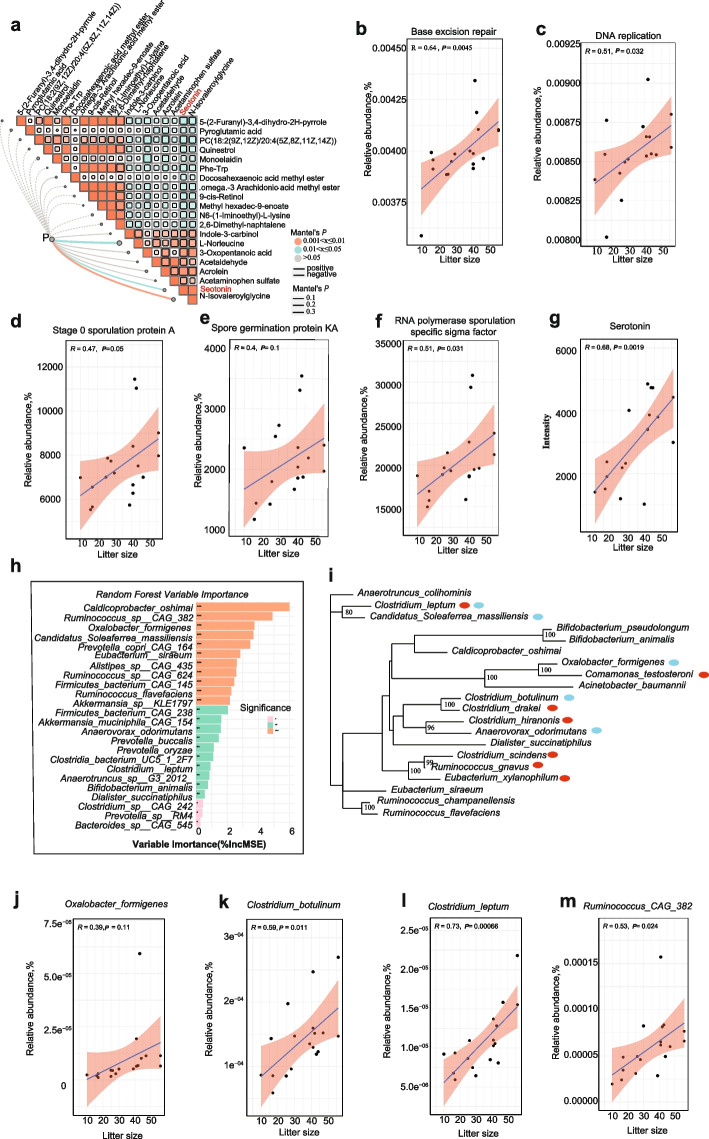
High fertility reshaped gut microbiota and key species related to serum serotonin. **a** Pairwise comparisons of differently abundant metabolites, with a color-gradient denoting Spearman’s correlation coefficients. Spore-related genes (P) based on network analysis are related to each taxon by partial Mantel tests. Edge width corresponds to the Mantel’s statistic for the corresponding distance correlations, and edge color denotes the statistical significance. **b–g** The correlations between base excision repair, DNA replication, key spore-related genes and serotonin with litter size. The shaded areas represent the 95% confidence intervals for predictions from a linear model. **h** The significant bacterial biomarkers identified by random forests regression of relative abundances of LEfSe-determined bacterial taxon (LDA > 2) against serum serotonin concentration. Statistical significance of selected bacterial biomarkers was assessed by permutation test (999 times), with * = pink (*P* < 0.05), ** = blue (*P* < 0.01), and *** = orange (*P* < 0.001). **i** Phylogenetic tree displaying key serotonin-related bacteria based on LEfSe-determined bacterial taxon (LDA > 2) (green circles) and relative to reference species with reported serotonin-related species (red circles). **j–m** The correlations between key serotonin-related bacteria and litter size. The shaded areas represent the 95% confidence intervals for predictions from a linear model

Given that all sows were reared under identical environmental conditions and received the same diet, it raises the question: What drives the variations in the intestinal microbiota among sows with differing reproductive performances? We hypothesized that the discrepancies in microbial communities among individuals are due to variations in litter size and associated health stress. This implies that a higher litter size prompts gut microbial remodeling to adapt to host physiological changes, alongside an upregulation of serotonin in high fertility sows (Fig. 8a). Subsequently, we undertook a comprehensive analysis to explore the associations between stress-related characteristics and the reproductive phenotypes of sows, specifically focusing on litter size. Correlation analysis demonstrated that several key KEGG pathways and spore formation genes, including base excision repair, DNA replication, stage 0 sporulation protein A, spore germination protein KA, and RNA polymerase sporulation-specific sigma factor, were positively associated with the litter size across all samples (Fig. 8b–g). The aforementioned analyses show associations between cumulative litter size and specific genes associated with sporulation.

To further pinpoint the key bacteria associated with sow serotonin levels, we analyzed the abundance of LEfSe-distinguished bacterial taxa against serum serotonin concentrations using the random forests machine learning algorithm. In the gastrointestinal (GI) tract, serotonin is synthesized by specialized endocrine cells, called enterochromaffin cells (ECs). Numerous studies have highlighted the association between gut microbes and host serum serotonin synthesis and release [5]. In our study, certain bacteria, such as *Caldicoprobacter oshimai*, *Ruminococcus* sp. CAG 382, *Candidatus Soleaferrea massiliensis*, *Oxalobacter formigenes*, and *Clostridium leptum*, were found to be associated with higher serotonin level (Fig. 8h). Given the crucial role of gut microbiota in regulating host serotonin and reproductive performance function, we further aimed to identify specific microbial species responsible for conferring the serotonergic effects. Phylogenetic analysis of metagenomic sequences revealed that a subset of microbes aligned with spore-forming bacteria-colonized mice cluster taxonomically with *Clostridia* [33]. Notably, there are striking phylogenetic commonalities between taxa identified as serotonin-related microbiota in spore-forming-colonized mice [35] and those in our study (including *Oxalobacter formigenes*, *Clostridum botulinum*, *Clostridum leptum*, *Candidatus Soleaferrea massiliensis*) (Fig. 8i). This similarity supports their role in promoting serotonin synthesis from colonic ECs. Having identified key microbes that may modulate host serotonin, we next investigated whether these microbes were positively correlated with the litter size of each sow, and the results further verified our hypothesis that a larger litter size could potentially induce host health stress and change gut microbial community composition (Fig. 8j–m).

## Discussion

The impact of gut microbiota on sex hormones and host metabolites has been well-documented in several studies [41, 42]. Observations have indicated changes in testosterone levels following the elimination of commensal gut microbiota in female mice. Women with greater gut microbiota diversity exhibited elevated ratios of hydroxylated estrogen metabolites to estradiol in their urine [43]. Additionally, systemic estrogens showed a strong positive correlation with fecal Clostridia taxa and specific genera within the Ruminococcaceae family [44]. This study found that bacteria related to serotonin may maintain reproductive performance of sows.

In this study, both PCoA plots generated from 16S rRNA gene sequencing and metagenomic sequencing illustrated distinct clusters between the two groups. This indicates notable alterations in the microbial community structure and composition among Jinhua sows with differing reproductive phenotypes (number of healthy piglets per litter). The microbial profiles of HRP and LRP sows exhibited disparities in the relative abundances of taxa across multiple taxonomic levels. Notably, the abundance of the Firmicutes, Bacteroidetes, and Spirochaetes phyla accounted for a large proportion of the sow gut microbiota in our study, consistent with previous studies [45]. Additionally, our study revealed no disparity in α-diversity, consistent with certain investigations [46], although other studies have indicated higher α-diversity in pigs with high productivity compared to those with low productivity under similar dietary conditions [47]. At the genus level, the variation of different taxa remains consistent between 16S rRNA gene sequencing and metagenomic sequencing, with *Clostridium* and *Ruminococcus* being enriched in the HRP group. These findings underscore the potentially pivotal roles of these bacteria in contributing to beneficial effects in the host [48]. Moreover, the elevated abundances of specific bacterial species (such as *Caldicoprobacter oshimai*, *Ruminococcus* sp. CAG 382, *Eubacterium siraeum*, *Candidatus Soleaferrea massiliensis*, *Oxalobacter formigenes*, *Clostridium leptum*, and *Bifidobacterium pseudolongum*) in HRP sows suggest that these microbes are likely key contributors to the number of the piglets. *Bifidobacterium pseudolongum*, widely present in the mammalian gut, is known for its abundance correlating significantly with animal health, such as enhancing immune responses and potentially influencing metabolic [49]. In terms of archaea, the increased relative abundance of the genus *Methanobrevibacter*, which utilizes carbon sources to produce methane, leads to energy wastage in high fertility sows, as the methane is emitted rather than being utilized by the microbes or the host [50], implying a heightened metabolic burden on both the host and its microbiota. The microbial networks of the top 100 abundances further reveal the differences between the two groups.

The observed difference in the gut microbiota could be attributed to intrinsic and extrinsic factors, such as age, host characteristics, health status, and diet [51, 52]. Diet has the potential to significantly alter the composition of the gut microbial community and influence the metabolism of both microbes and the host. Given that the Jinhua sows were under uniform dietary conditions and management, with comparable dietary habits across our cohorts, it is reasonable to speculate that intrinsic factors are primarily responsible for the observed alterations in the composition and functions of the gut microbiota. This speculation is further supported by the consistent enrichment of base excision repair, DNA replication, and spore-related genes in HRP sows, as revealed by metagenomic analysis [37]. To our knowledge, base excision repair is a major pathway for the removal of endogenous and exogenous DNA damage caused by environmental stress [53–55]. Additionally, the upregulation spore formation suggests that the gut microbiota is adapting to environmental stress [56, 57]. Correlation analyses between base excision repair, DNA replication, and key spore-related genes with litter size further underscore the sows’ contribution to gut microbiota dynamics. This is particularly significant given that previous studies have shown that more offspring impose a substantial metabolic burden on women [58]. In our study, the metabolic burden on HRP sows, particularly in amino acid and energy metabolism (Fig. 5e), caused by a larger litter size (Fig. 8), may impact the mutualistic symbiosis between the host and its gut microbes, potentially allowing for microbial adaptation.

As discussed, gut microbes can significantly influence the biosynthesis of reproductive and steroid hormones, thereby affecting reproductive capacity. Hormones such as follicle-stimulating hormone (FSH), luteinizing hormone (LH) and progesterone play crucial roles in ovarian folliculogenesis, gonadal function, and regulating the menstrual cycle, pregnancy, and aspects of sexuality, directly impacting the total litter size [59–61]. However, despite the importance of these hormones, this study revealed no significant differences in these critical hormones according to metabolomics analysis (Table S2). Intriguingly, the findings indicate elevated blood serotonin levels in sows with high reproductive performance. It is noteworthy that over 90% of the body’s serotonin is synthesized by tryptophan hydroxylase (TPH1), which converts tryptophan in endocrine cells. Serotonin plays a pivotal role in linking microbiota with various biological effects, including enteric motor functions, secretory reflexes, immune responses, platelet aggregation, bone development, cardiac function, and reproductive maintenance [62–65]. It is also known to stimulate Aryl hydrocarbon receptors (AhRs) in immune cells, which may contribute to immune changes during pregnancy and farrowing. Additionally, serotonin and its receptors have been identified in the mammalian oviduct, uterus, and ovaries [66–68]. For instance, serotonin receptor signaling may affect ovarian granulosa cells via the Bcl-2 gene family, a mechanism that could be crucial for ovarian development [69, 70]. Additionally, research indicates that serotonin signaling modulates the sexual receptivity of virgin female *Drosophila* [71]. These findings highlight the broad and critical roles serotonin plays in regulating both immune responses and reproductive functions. In our study, multi-omics correlation analysis revealed a positive correlation between serotonin levels and both the number of healthy piglets per litter and total piglets per sow. These results suggest beneficial potential effects of serotonin on ovarian follicle survival and oocyte maturation [72].

Previous research has identified gut microbiota as a master regulator of serotonin production and release in the GI tract, maintaining serotonin homeostasis and facilitating its distribution throughout various body sites [73, 74]. In this study, we demonstrated that the gut microbiota may influence host reproductive phenotypes, particularly litter size, by affecting the host’s serotonin levels. Notably, the random forest machine learning algorithm revealed that enriched bacteria taxa in HRP sows were associated with host serotonin. Further studies are required to integrate specific key bacteria in our study, as conducted by phylogenetic tree displaying, according to other studies. For instance, it was determined that the bacterium *Ruminococcus gnavus* plays a critical role in serotonin production [75], Monocolonization of germ-free mice with *Ruminococcus gnavus* can stimulate the production of peripheral serotonin. *Ruminococcus gnavus*-mediated catabolism of dietary phenylalanine and tryptophan generated phenethylamine and tryptamine that directly stimulated serotonin biosynthesis in intestinal ECs via a mechanism involving activation of trace amine-associated receptor 1 (TAAR1) [75]. Additionally, another study demonstrated that indigenous spore-forming microbes from the colons of mice and humans significantly enhance microbiota effects on colonic and blood serotonin levels [76]. These findings collectively underscore the important role of gut microbiota in modulating serotonin levels and its potential implications for reproductive phenotypes.

The alignment of microorganisms associated with serotonin levels, belonging to the genera *Clostridium* and *Ruminococcus*, with those enriched in HRP group is noteworthy. There are striking phylogenetic commonalities between species enriched in HRP sows consistent with discovered gut microbiota related to stimulation of serotonin synthesis in intestinal cells. Specifically, *Oxalobacter formigenes* resembles *Comamonas testosteroni*, *Clostridium botulinum* resembles *Clostridium drakei*, and *Anaerovorax odorimutans* resembles *Clostridium hiranonis*, suggesting that these microorganisms may share similar functions in stimulation of serotonin synthesis. Furthermore, *Clostridium leptum* was found to be upregulated in the HRP group, and it has been reported to stimulate serotonin production [75]. This is an exciting association because each of these species, in accordance with the previous report [75], have been linked to litter size.

Collectively, these findings reinforce our hypothesis that increased litter sizes may impose significant metabolic, reproductive, and health burdens on sows, consequently prompting changes in the microbial community and its function in stimulating serotonin production. Previous studies have suggested that gut microbiota can engage in interactions with their hosts, fostering co-evolution and leading to the adaptation [4]. This interaction may play a critical role in influencing gut microbes of HRP sows. Indeed, our findings indicate an enrichment in the abundance of these serotonin-related species, such as *Oxalobacter formigenes*, *Ruminococcus* sp. CAG 382, *Clostridium leptum*, and *Clostridium botulinum*, which contributed to maintaining excellent reproductive performance in sows under this examination. Further extensive research is needed to thoroughly investigate the identified serotonin-related microbiota and its potential impact on serotonin levels in mice, to validate and expand upon these findings. Uncovering more specific bacteria, particularly those associated with serotonin, in this area could significantly advance our comprehension of the mechanisms by which gut microbiota influence swine production.

## Conclusions

To our understanding, this investigation marks the foray into the amalgamation of gut microbiota and host metabolome profiles in a Jinhua sow model displaying diverse reproductive performance, aiming to delineate their intricate interconnections. The comprehensive analysis revealed clear distinctions in microbiota and metabolic profiles (encompassing structure, composition, and function) among sows with varying reproductive performance. Intriguingly, our research revealed variations in intestinal microbiota that correlated with the litter size of the sows. These variations coincided with an increase in serotonin-related bacteria, potentially influencing the reproductive performance of host. KEGG pathway analysis of the microbiota data indicated an enrichment of base excision repair, DNA replication, and spore-related genes in sow with high reproductive performance, implying that the gut microbiota is reassembled influenced by stress. Subsequent analyses demonstrated a positive correlation between the functions involved in base excision repair, DNA replication, and genes of spore formation and total litter size, suggesting reassembling gut microbiota in sows with higher litter sizes. Notably, species such as *Oxalobacter formigenes*, *Anaerovorax odorimutans*, *Clostridium leptum*, *Clostridium botulinum*, and *Ruminococcus* sp. CAG 382 may play crucial roles within the microbiota, contributing to elevated serotonin levels. Multi-omics correlation analysis revealed a positive correlation between serotonin levels with the number of healthy piglets per litter and total piglets per sow. This study outlines the role of gut microbiota, particularly through probiotic mechanisms, in regulating reproductive performance, thereby establishing a foundation for understanding potential mechanisms underlying human reproductive disorders.

## Supplementary Information

Additional file 1: Fig. S1. The fecal microbiota of HRP and LRP sows using 16S rRNA gene amplicon sequencing. Fig. S2. Fecal microbiota of HRP(*n=*10) and LRP sows (*n=*8) can be distinguished using metagenome sequencing.

Additional file 2: Table S1. The reproductive performance of sows in our study. Table S2. The result of fecal metabolomics. Table S3. The relative abundance of genes related pore formation. Table S4. The *P *value of enrichment pathway about significantly different metabolites. Table S5. The information of networks in the LRP group groups. Table S6. The information of networks in the HRP group groups. Table S7. The detail of correlation analysis.

## Data Availability

16S rRNA gene amplicon sequencing data of are deposited under NCBI BioProject accessions PRJNA1118910 accessions. Metagenomic sequencing data of are deposited under NCBI BioProject accessions PRJNA1119245. Metabolomics data are deposited in MetaboLights with number MTBLS10332.

## Abbreviations

CAZyme: Carbohydrate-active enzyme
EC: Enterochromaffin cell
FC: Fold change
GI: Gastrointestinal
HRP: High reproductive performance
KEGG: Kyoto Encyclopedia of Genes and Genomes
LC-MS: Liquid chromatography mass spectrometry
LDA: Linear discriminant analysis
LEfSe: Linear discriminant analysis effect size
LRP: Low reproductive performance
MRP: Medium reproductive performance
MS2: Mass spectrometry 2
NCBI: National Center for Biotechnology Information
OPLS-DA: Orthogonal partial least squares discriminant analysis
PCA: Principal component analysis
PCoA: Principle coordinate analysis
PCOS: Polycystic ovary syndrome
SCFA: Short-chain fatty acid
TPH1: Tryptophan hydroxylase
VIP: Variable importance in projection
