# Supplementary material for: High-fertility sows reshape gut microbiota: the rise of serotonin-related bacteria and its impact on sustaining reproductive performance
Source: J Anim Sci Biotechnol. 2025 May 22;16:73. doi: 10.1186/s40104-025-01191-z (PMC12096716; doi:10.1186/s40104-025-01191-z)
Supplement: Supplementary file 1 — Additional file 1: Fig. S1. The fecal microbiota of HRP and LRP sows using 16S rRNA gene amplicon sequencing. Fig. S2. Fecal microbiota of HRP(n=10) and LRP sows (n=8) can be distinguished using metagenome sequencing. [file 40104_2025_1191_MOESM1_ESM.docx]

**Supplementary Figures**

**Additioanl file 1**


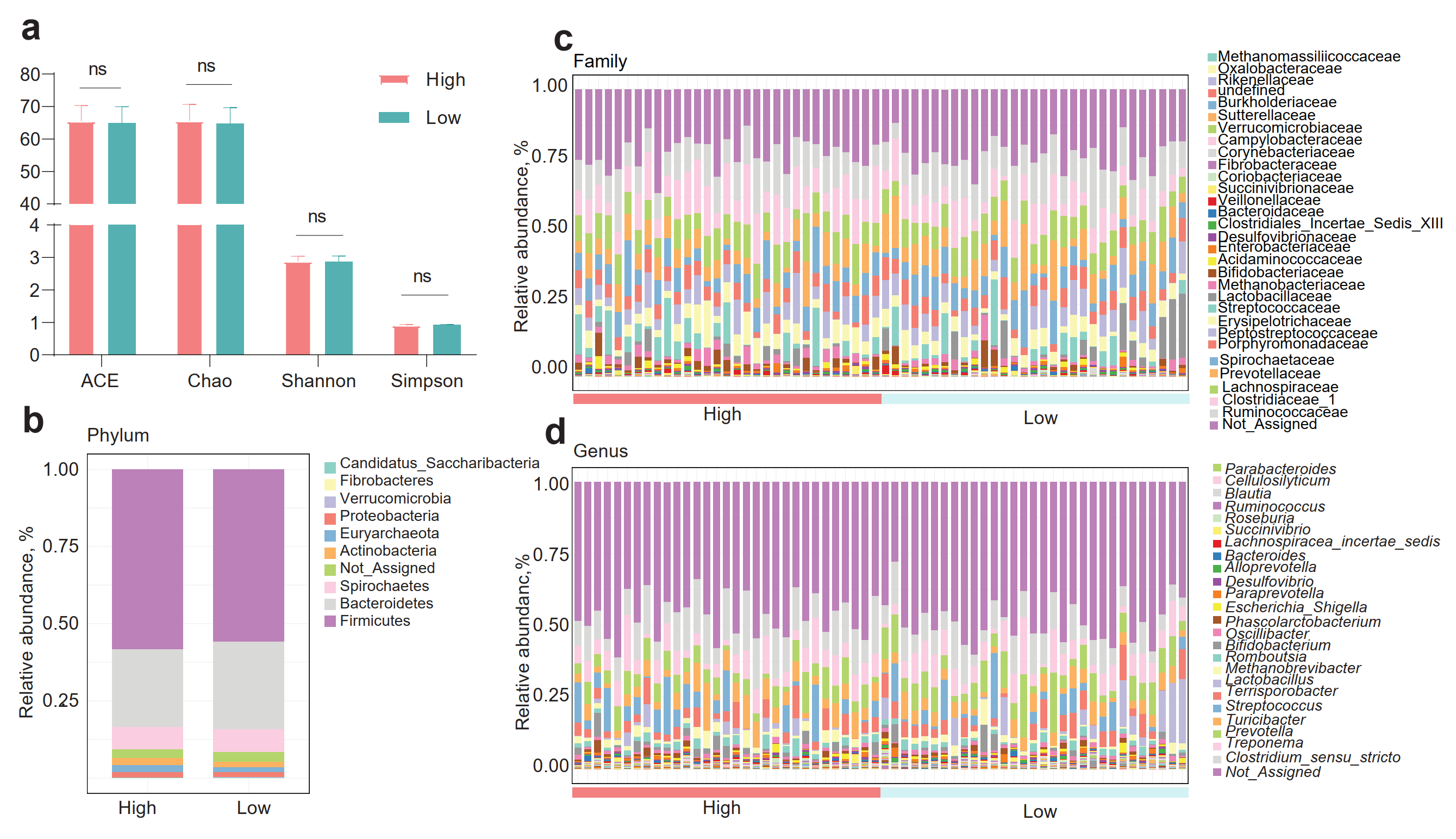


**Fig. S1** The Fecal microbiota of HRP and LRP sows (*n* = 31) using 16S rRNA gene amplicon sequencing. (**a**) The α-diversity of HPR and LPR sows measured using ACE, Chao, Shannon and Simpson indices. (**b**) The relative abundance of microbial composition at the phylum level. (**c** and **d**) The relative abundance of microbial composition at the family and genus level


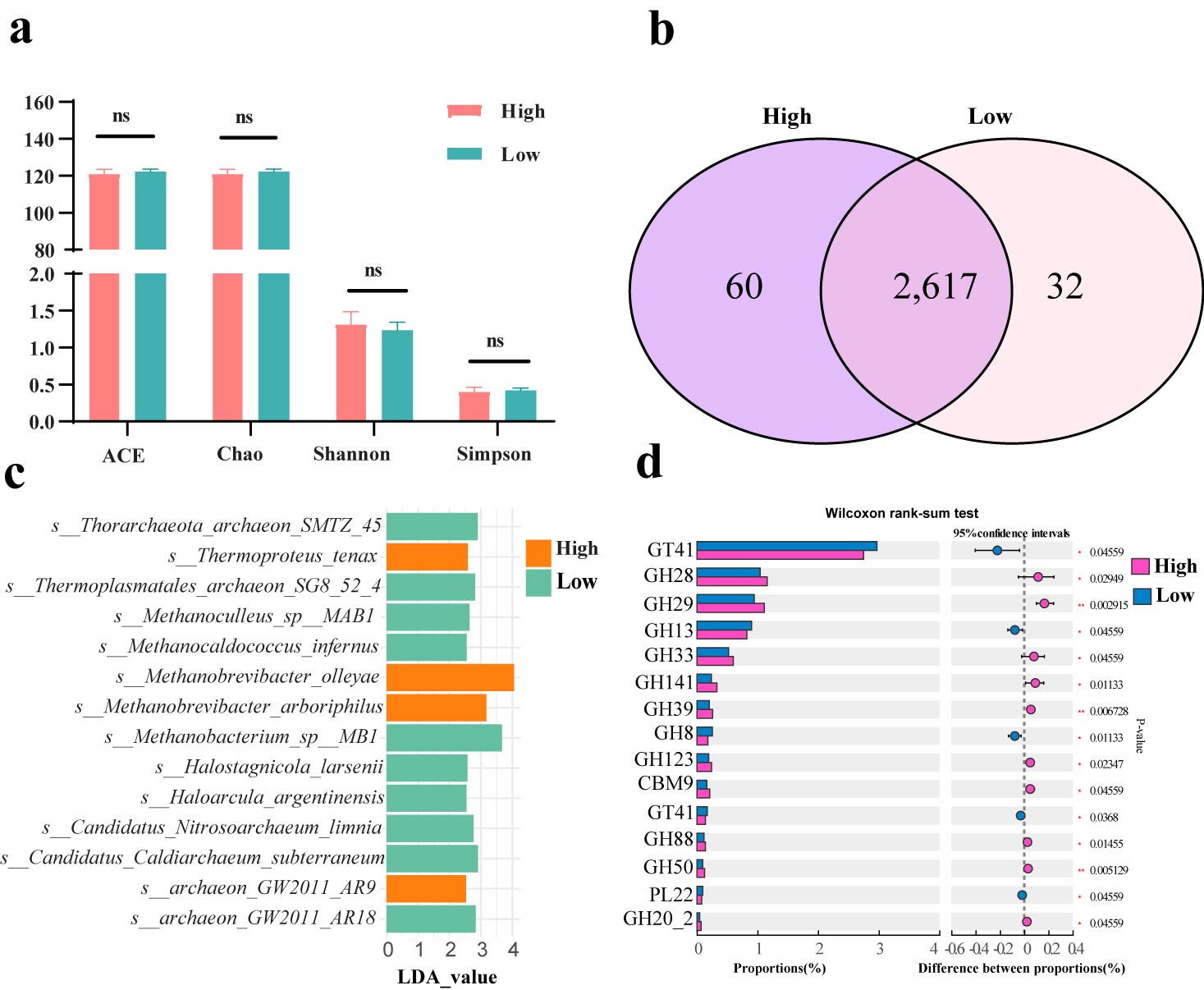


**Fig. S2** Faecal microbiota of HRP(*n*=10) and LRP sows (*n* =8) can be distinguished using metagenome sequencing. (**a**) The α-diversity of HPR and LPR sows measured using ACE, Chao, Shannon and Simpson indices. (**b**) Venn diagram analysis based on different groups of gut microbes on genu level. (**c**) LDA score plot of enriched archaea taxa abundance determined by linear discriminant analysis effect size (LEfSe) analysis (LDA value>2.0; *P*-value < 0.05. (**d**) Differential CAZyme functions in the two groups based on family-level enzymes (Wilcoxon rank-sum test, *P* < 0.05). CBM, carbohydrate‐binding modules; GH, glycoside hydrolase family; GT, glycosyl transferase; PL, polysaccharide lyase
